# Diffusion MRI in prostate cancer with ultra-strong whole-body gradients

**DOI:** 10.1002/nbm.5229

**Published:** 2024-08-27

**Authors:** Malwina Molendowska, Marco Palombo, Kieran G. Foley, Krishna Narahari, Fabrizio Fasano, Derek K. Jones, Daniel C. Alexander, Eleftheria Panagiotaki, Chantal M.W. Tax

**Affiliations:** 1 Cardiff University Brain Research Imaging Centre (CUBRIC), Cardiff University, Cardiff, UK; 2 School of Computer Science and Informatics, Cardiff University, Cardiff, UK; 3 Division of Cancer and Genetics, School of Medicine, Cardiff University, Cardiff, UK; 4 Cardiff and Vale University Health Board, Heath Park Campus, Cardiff, UK; 5 Siemens Healthcare Ltd, Camberley, UK; 6 Siemens Healthcare GmbH, Erlangen, Germany; 7 Centre for Medical Image Computing, University College London, London, UK; 8 Image Sciences Institute, University Medical Center Utrecht, Utrecht, The Netherlands

**Keywords:** diagnostic imaging, diffusion MRI, high *b*-values, prostate cancer, strong gradients

## Abstract

Diffusion-weighted MRI (dMRI) is universally recommended for the detection and classification of prostate cancer (PCa), with PI-RADS recommendations to acquire *b*-values of ≥1.4 ms/μm^2^. However, clinical dMRI suffers from a low signal-to-noise ratio (SNR) as the consequence of prolonged echo times (TEs) attributable to the limited gradient power in the range of 40−80 mT/m. To overcome this, MRI systems with strong gradients have been designed but so far have mainly been applied in the brain. The aim of this work was to assess the feasibility, data quality, SNR and contrast-to-noise ratio (CNR) of measurements in PCa with a 300 mT/m whole-body system. A cohort of men without and with diagnosed PCa were imaged on a research-only 3T Connectom Siemens MRI system equipped with a gradient amplitude of 300 mT/m. dMRI at high *b*-values were acquired using high gradient amplitudes and compared with gradient capabilities mimicking clinical systems. Data artefacts typically amplified with stronger gradients were assessed and their correction evaluated. The SNR gains and lesion-to-healthy tissue CNR were statistically tested investigating the effect of protocol and *b*-value. The diagnostic quality of the images for different dMRI protocols was assessed by an experienced radiologist using a 5-point Likert scale and an adapted PI-QUAL scoring system. The strong gradients for prostate dMRI allowed a significant gain in SNR per unit time compared with clinical gradients. Furthermore, a 1.6−2.1-fold increase in CNR was observed. Despite the more pronounced artefacts typically associated with strong gradients, a satisfactory correction could be achieved. Smoother and less biased parameter maps were obtained with protocols at shorter TEs. The results of this study show that dMRI in PCa with a whole-body 300-mT/m scanner is feasible without a report of physiological effects, SNR and CNR can be improved compared with lower gradient strengths, and artefacts do not negate the benefits of strong gradients and can be ameliorated. This assessment provides the first essential step towards unveiling the full potential of cutting-edge scanners, now increasingly becoming available, to advance early detection and diagnostic precision.

## Abbreviations

ADC: apparent diffusion coefficient
AP: anterior−posterior
AS: Active Surveillance
CNR: contrast-to-noise ratio
dMRI: diffusion-weighted magnetic resonance imaging
EPI: echo planar imaging
GS: Gleason score
MD: mean diffusivity
MK: mean kurtosis
mp-MRI: multiparametric MRI
PA: posterior−anterior
PCa: prostate cancer
PGSE: pulsed gradient spin echo
PI-RADS: prostate imaging-reporting and data system
PSA: prostate-specific antigen
PZ: peripheral zone
ROI: region of interest
TRUS: transrectal ultrasound scan
VERDICT: vascular, extracellular and restricted diffusion for cytometry in tumours

## 1 Introduction

Diffusion-weighted magnetic resonance imaging (dMRI)^1^ sensitises the MRI signal to the random motion of water molecules by applying diffusion-encoding gradients. Tissue-water mobility is affected by histological features, such as the density of cells and their size, shape and arrangement. As a consequence, dMRI has gained recognition in cancer detection, diagnosis and monitoring.

The apparent diffusion coefficient (ADC)—derived from data with moderate diffusion weightings (e.g., *b*-value < 1 ms/μm^2^) at a fixed diffusion time—has been used in multiparametric MRI (mp-MRI) protocols as a sensitive quantitative measure for microstructural changes in cancer. Although promising, the required diffusion weighting for precise quantification of ADC necessitates the application of diffusion encoding gradients for several tens of milliseconds using clinical systems, and the consequent long echo times (TEs) culminate in a low signal-to-noise ratio (SNR) because of additional signal loss from transverse relaxation (*T*_2_). In addition, a variation in ADC cannot be specifically attributed to different aspects of the pathology that alter the classification of the tumour.^2^ Simultaneous changes in the properties of different microscopic compartments may even result in no net alteration of ADC potentially causing cancerous lesions to not always be visible on mp-MRI.^3^

**Figure 1:**
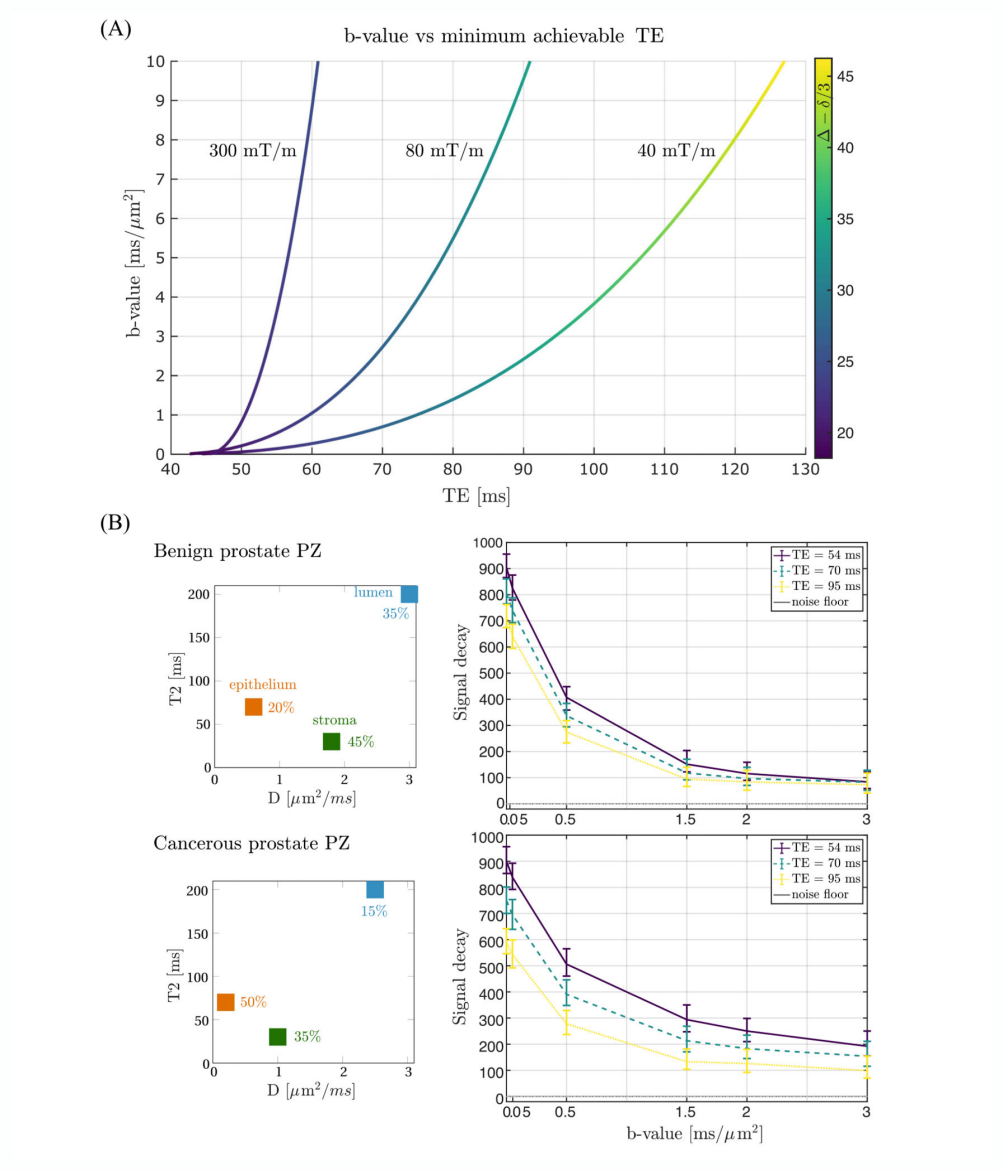
(A) Simulation of *b*-value versus minimum achievable TE in SE EPI (1.3 × 1.3 mm^3^, partial Fourier = 6/8, GRAPPA = 2, FOV = 220 × 220 mm^2^) experiment with different gradient system configurations: 300 mT/m/111 T/m/s, 80 mT/m/200 T/m/s and 40 mT/m/200 T/m/s for diffusion encoding along a single scanner axis. For each point, the colour coding gives the diffusion time achieved (Δ - δ/3 [ms]). (B) (*left*) The prostate gland is highly heterogenous, composed of densely packed cellular compartments (stroma and epithelium) and ‘waterlakes’ (lumen), which exhibit different diffusion and relaxation properties. Diagram of a *T*_2_ − *D* spectrum in the PZ in benign tissue and malignant adenocarcinoma (GS 3 + 4)^7^ with three pools: stroma and epithelium (cellular compartments of anisotropic and restricted diffusion characteristics with short *T*_2_s) and lumen (unrestricted diffusion with long *T*_2_). (*right*) Simulation of the corresponding signal resulting from the three-pool model (median with interquartile range) for three TEs defined by the maximum gradient strength available (300, 80 and 40 mT/m). The simulation details are given in the Supporting Information (Section SI1).

Higher *b*-value dMRI (*b* > 1 ms/μm^2^) can improve the lesion visibility,^4^ which, in turn, could facilitate its detection/delineation^5,6^ and the separation of signal contributions from different compartments^7^ (Figure 1B). Indeed, a recent clinical trial has shown that a proxy for intracellular signal contributions^8^ can improve the classification of clinically significant cancer compared with ADC,^9^ potentially providing a ‘virtual biopsy’ and avoiding unnecessary invasive tests. However, high *b*-value dMRI is challenging to acquire using clinical gradient systems, as even longer diffusion encoding gradients are required. Alternatively, mp-MRI protocols predict high *b*-value images from moderate *b*-value images, but these images essentially do not contain novel information, and high *b*-value signals can deviate significantly from predictions from lower *b*-value signals.^10^

The solution to obtaining high *b*-values lies in the use of stronger gradients for diffusion encoding. Several ultra-strong gradient systems have been designed,^11^ providing shorter TE for a given *b*-value, and hence higher SNR, that is, reduced signal loss due to *T*_2_ (Figure 1A). These technological advances have greatly facilitated the characterisation of tissue microstructure.^12–14^ Nevertheless, ultra-strong gradient systems have almost exclusively been used for imaging of the brain and only recently also below the neck in healthy volunteers.^15,16^

This work shows the first results of dMRI in the healthy and cancerous prostate on a scanner equipped with 300 mT/m gradients—the strongest gradient strength available for whole-body imaging that was originally only released for brain imaging. Cancerous lesions typically show a greater signal contribution from compartments with a larger degree of diffusion restriction, and the predicted increase in signal from the use of strong gradients in the prostate is significant (Figure 1B).

The goal of this manuscript is to explore the remaining signal at increasingly higher *b*-values, which are off-limits with clinical hardware because of the necessary long TEs (in line with PI-RADS recommendations of acquiring a *b*-value of ≥1.4 ms/μm^2^), and to assess and correct for artefacts common in dMRI that may be amplified with strong gradients. Specifically, this work focuses on assessing data quality, SNR and contrast-to-noise ratio (CNR) of measurements at high gradient strengths compared with clinically available strengths, which is the first essential step towards unveiling the full potential of cutting-edge scanners, now increasingly becoming available. Ultimately, the new and unexplored signal regime at high *b*-values and short echo times could provide a higher lesion-to-healthy-tissue contrast and improved characterisation of tissue microstructure with multi-compartment models.

## 2 Methods

### 2.1 Study population

Ethical approval was obtained from the School of Psychology Research Ethics Committee (REC) of Cardiff University and from the National Health Service (NHS) REC (ref 20/OCT/8264).

Four healthy men (age range: 46−64 years, mean [*M*] = 54.8 years, standard deviation [*SD*] = 7.9 years, weight range: 75−104 kg, *M* = 85.6 kg, *SD* = 12.7 kg, height range: 1.68−1.81 m, *M* = 1.76 m, *SD* = 0.06 m) and six patients diagnosed with prostatic adenocarcinoma enrolled in the Active Surveillance Programme (age range: 67−74 years, *M* = 70.2 years, *SD* = 2.9 years, weight range: 75.3−100.0 kg, *M* = 85.5 kg, *SD* = 9.2 kg, height range: 1.72−1.81 m, *M* = 1.76 m, *SD* = 0.04 m) with peripheral zone (PZ) prostate cancer confirmed by elevated prostate-specific antigen (PSA), MRI and transrectal ultrasound scan (TRUS) guided biopsy (with Gleason score 3 + 3 or 3 + 4) were included in the study, after MRI safety screening and written consent. Detailed patient cohort information is provided in Table 1. The participants were advised to follow a low residue diet for 24 h prior to imaging to reduce the risks of artefacts in the data. The key inclusion criteria for the clinical population were (1) no recent biopsy (<6 months) or previous radiation treatment and (2) a visible lesion of prostate cancer in PZ on conventional MRI with a positive histopathological correlation of prostate cancer biopsy.

### 2.2 Data acquisition

Imaging data were acquired on a Connectom research-only MRI scanner, a modified 3T MAGNETOM Skyra system equipped with a gradient coil capable of 300 mT/m (Siemens Healthcare, Erlangen, Germany) using two surface coils (18-channel body coil and 32-channel spine coil).

Structural MRI data were acquired using a 3D *T*_2_-weighted turbo spin echo (TSE) sequence in the axial plane with parameters: TE = 102 ms, TR = 1920 ms, isotropic voxel = 1 mm^3^, number of slices = 72, in-plane FOV = 230 × 230 mm^2^, phase encoding (PE) direction = right−left, with varying flip angle (due to different limitations of SAR between subjects). The total acquisition time was 10 min.

Diffusion-weighted images were acquired using a research application pulsed gradient spin echo (PGSE) echo planar imaging (EPI, single-shot with *G*_max_ = 39 mT/m, SR = 186 T/m/s for readout) sequence.^1^ Six shells of 15 non-collinear directions distributed on a sphere at diffusion weightings of *b* = [0, 0.05, 0.5, 1.5, 2, 3] ms/μm^2^ were sampled with non-diffusion-weighted *b* = 0 ms/μm^2^ images interspersed, all with anterior-posterior PE direction. The order of acquisition was randomised to lower the thermal load of the scanner.^17^ dMRI protocols were designed to reflect diffusion-encoding gradient strengths common on high performance (*G*_max_ = 273 mT/m, at SR = 110 T/m/s; here, the maximum SR is limited because of safety constraints, i.e., PNS and cardiac stimulation^18,19^) and clinical systems (*G*_max_ = 40 or 80 mT/m, at SR = 200 T/m/s). Note that the readout was the same across protocols. The minimum achievable TE, δ and Δ were as follows (TE/δ/Δ [ms]): (P1, where P stands for protocol) 54/5/25, (P2) 70/16/32 and (P3) 95/26/48, for a maximum achievable gradient strength of 300, 80 and 40 mT/m, respectively. TR was constant across protocols and set to 3.5 s, to harmonise longitudinal relaxation effects. The in-plane resolution was 1.3 × 1.3 mm^2^, FOV = 220 mm × 220 mm^2^, matrix size = 168 × 168, slice thickness = 5 mm, slices = 14, GRAPPA = 2, simultaneous multislice = 2, partial Fourier = 6/8 and bandwidth of 1860 Hz/pixel. Additionally, three non-diffusion weighted images with posterior-anterior PE direction were acquired. The total acquisition time of the three protocols (in AP and PA PE directions) was approximately 20 min (6 min 35 s per protocol).

Furthermore, in two patients, a set of three protocols (P4−P6) with increased maximum *b*-values were designed (additional *b*-values were *b* = [3.5, 4, 4.5, 5] ms/μm^2^) to further investigate the signal decay in lesions. The achievable TE/δ/Δ ([ms]) were as follows: (P4) 56/6/26, (P5) 79/20/35 and (P6) 106/34/50, for a maximum achievable gradient strength of 300, 80 and 40 mT/m, respectively, with the remaining parameters as described above. The total acquisition time of these additional three protocols was 28 min. dMRI data were reconstructed using an ‘adaptive combine’ option with an optimal phase shift factor = 4. Data processing methods implemented by the vendor, apart from ‘prescan normalise’, were switched off.

In addition, a clinical-like dMRI scan was run using a vendor’s product sequence with default parameters (*b*-values = [0, 0.1, 0.4, 0.8] ms/μm^2^, each in three directions with 8 repetitions, TE = 61 ms, TR = 3.5 s, δ = 8.4 ms, Δ = 28 ms, in-plane resolution = 1.6 × 1.6 mm^2^, FOV = 260 mm x 260 mm^2^, matrix size = 160 × 160, slice thickness = 3.3 mm, slices = 20, GRAPPA = 2, no simultaneous multislice, partial Fourier = 6/8 and bandwidth of 1202 Hz/pixel; raw and dynamic distortion correction filters were on). An image at *b*-value = 1.4 ms/μm^2^ was extrapolated by the vendor’s reconstruction programme. The total acquisition time was 5 min.

### 2.3 Quality assessment and data preprocessing

The reconstructed dMRI data were quality-checked.^20^ The noise standard deviation was estimated based on low *b*-value magnitude data up to 1.5 ms/μm^2^ ^21^ to minimise Rician bias in the noise estimate. SNR was calculated on the unprocessed data for P1−P3 as $\text{SNR}=\frac{{\tilde{S}}_{ROI}}{{\tilde{\sigma }}_{ROI}},$ where ${\tilde{S}}_{ROI}$ is the median signal over all voxels across repetitions for each of the *b*-value and within a mid-gland slice mask (delineated on the average *b* = 0 ms/μm^2^) and ${\tilde{\sigma }}_{ROI}$ is the median of the estimated noise standard deviation of the noise map within the same mask. This procedure provides an SNR estimate of an individual image.

The data were denoised,^21^ Gibbs ringing corrected,^22^ distortion corrected (*B*_0_ field inhomogeneity^23^ and gradient non-uniformities^24^), and effective *b*-values and diffusion-encoding directions were computed^25^ (software: MRtrix^26^ version 3.0.2, FSL release 6.0,^27^ or in-house scripts). Neither eddy-current distortion correction nor motion correction was applied.

### 2.4 Image analysis

For qualitative analysis, powder-averaged signals were calculated. For the comparison of quantitative measures, mean diffusivity (MD) and mean kurtosis (MK) maps were derived from estimation of diffusion kurtosis imaging (DKI)^28^ (up to *b* = 2 ms/μm^2^) and accounting for deviations of the *b*-values and diffusion gradient directions.

Regions of interest (ROIs) were manually delineated in the dMRI images at either 1.5 or 2 ms/μm^2^, after confirming the lesion location with a radiologist. ROIs in tumour lesions (up to three ROIs per lesion, 10 ROIs in total) were delineated using a high *b*-value image from the protocol preferred by the radiologist (either P1 or P4), all in the PZ. Additionally, ROIs were drawn in normal-appearing PZ regions (up to three ROIs per subject, nine ROIs in total), for example, the contralateral side of the PZ with no tumour at the corresponding location in histology. From each ROI, signal values at *b* = 0.5−3 ms/μm^2^ were extracted. Two sets of data were defined: cancerous tissue (patients with GS 3 + 3 and 3 + 4 pulled together) and normal-appearing prostate tissue. The extracted values were averaged per ROI, and CNR was calculated as the within-subject difference between the mean signal from the lesions and healthy tissue, with the mean of the noise standard deviation within prostate as normalisation factor.

### 2.5 Statistical testing

To assess the effect of protocol on SNR, a linear mixed-effects model was employed with subject and *b*-value as random effects. Subsequently, a second linear mixed-effects model was utilised to evaluate the influence of the protocol within each specific *b*-value, with multiple comparison corrections (Bonferroni). Final pairwise comparisons were performed using the Wilcoxon test for non-parametric paired values, with correction for multiple comparisons (Bonferroni).

To assess the effect of protocol on the CNR, the metric was statistically tested w.r.t the effect of protocol across *b*-values and within each *b*-value.

### 2.6 Image diagnostic utility evaluation

The images acquired with P1−P3 were evaluated by a radiologist to investigate whether the images acquired with a high *b*-value with strong gradients improve the visibility and delineation of the lesions. The following images were presented simultaneously, in agreement with clinical practice: *T*_2_-weighted image and a set of dMRI images acquired with P1, P2 or P3, including average *b* = 0 ms/μm^2^ image, average at *b*-values = [0.5,1.5, 2] ms/μm^2^ and MD images. For patients scanned with P1−P3, we obtained 45 combinations (5 subjects × 3 protocols × 3 non-zero *b*-value images). The order of these combinations was randomised within each subject. Each combination was evaluated on its diagnostic quality with the following scoring: 1 below the minimum standard of diagnostic quality, 3 sufficient diagnostic quality and 5 optimal diagnostic quality. We adopted and modified the PI-QUAL system proposed by Giganti et al. (2020, 2021)^29^ to assess diagnostic value of the images, referred to as the ‘Quality index’. The radiologist was not informed about GS but was aware that the included patients were on Active Surveillance.

Furthermore, each set of images was subjectively assessed using a 5-point Likert image quality scale with respect to distortions and zonal anatomy. ‘Distortions’ (1 severe, 2 significant, 3 moderate, 4 low and 5 no influence) were defined as the general presence of artefacts and ‘zonal anatomy’ (1 poor, 2 below average, 3 average, 4 above average and 5 excellent) was defined as the ability to distinguish prostate zones. Finally, the radiologist was asked to select the preferred dMRI scan for each patient among P1−P3 in a side-by-side comparison (single slice data at all *b*-values), without knowledge of the protocol details.

## 3 Results

### 3.1 Quality assessment and preprocessing

Slice-wise outlier profiles revealed only few signal dropouts. Subtle shot-to-shot misalignments of the *b* = 0 ms/μm^2^ images were observed in protocols with the highest gradient strength (P1 and P4), presumably due to short- and long-term eddy currents and/or frequency drift (Section SI2 in the Supporting Information). For volumes acquired with non-zero diffusion weighting and sufficient tissue contrast, for example, *b* < 1.5 ms/μm^2^, minor misalignment to the first *b* = 0 ms/μm^2^ volume image could be observed due to EC.

**Figure 2:**
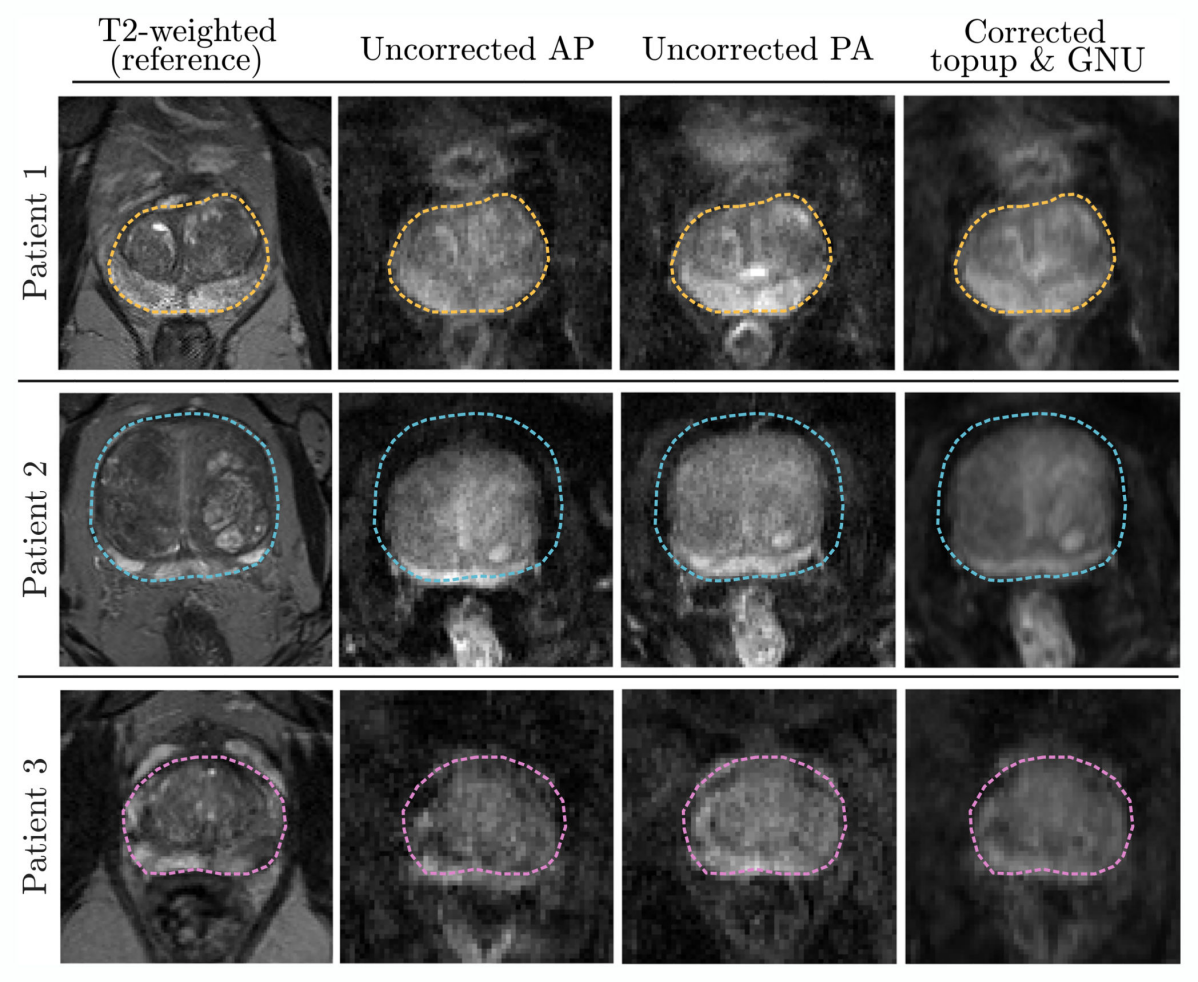
Visualisation of the distortion correction processing steps applied to the acquired dMRI data (in parenthesis, slice distance from isocentre is provided): Patient 1, ideally positioned at isocentre (0 cm); Patient 2, positioned off-centre (−10 cm); Patient 3, positioned at isocentre, with perceptually more severe distortions (0 cm). The reference *T*_2_-weighted images with delineated prostate gland (*dashed lines*) are shown (*the leftmost column*), alongside the phase-encoded in the AP and PA direction *b* = 0 ms/μm^2^ image from dMRI EPI scans (*2nd and 3rd column*). The applied corrections (*the rightmost column*) significantly improve the spatial correspondence between the *T*_2_-weighted and dMRI images.

Figure 2 shows the results of the preprocessing in three patient datasets, each affected by geometric distortions to varying degrees. After correction for susceptibility differences and gradient non-uniformities, geometrical discrepancies with respect to the *T*_2_-weighted scan were generally improved, for example, ameliorated signal pileups and restoration of spatial signal locations. The effects of gradient non-uniformities on the *b*-matrices are summarised in Supporting Information SI2, showing a *b*-value deviation from the imposed values by maximally 5% and typically in the range of 0−3% when the patient is placed at isocentre.

**Figure 3:**
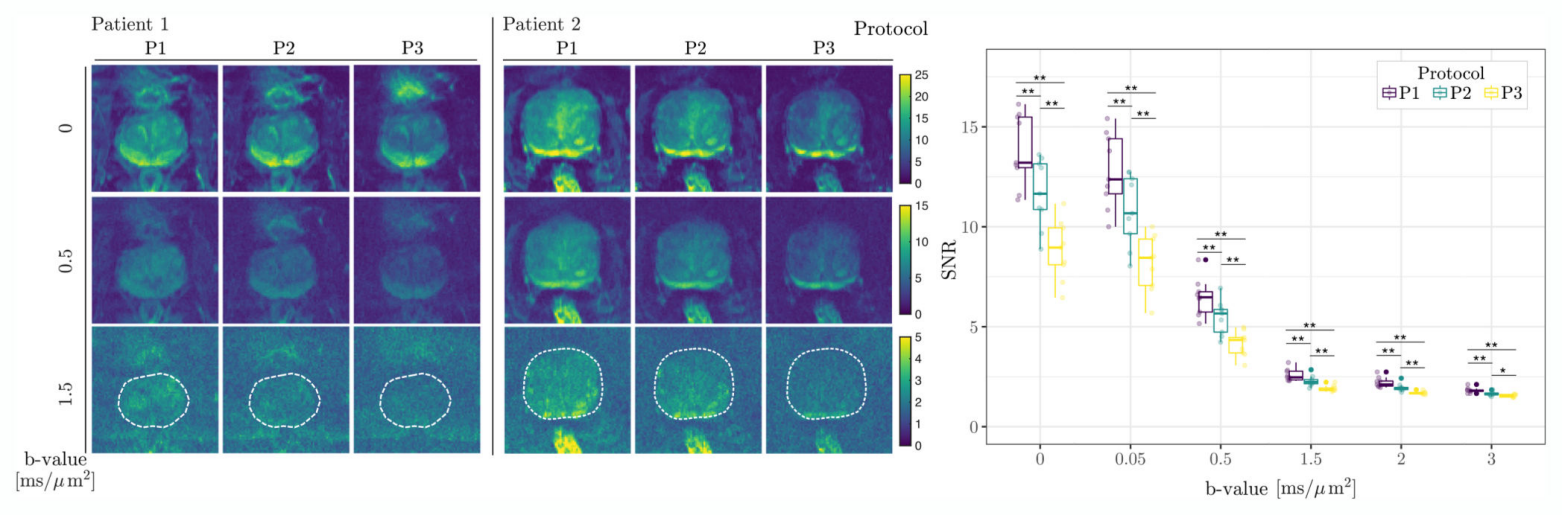
SNR analysis. (*left*) Spatial distributions of the SNR values for two representative patients and three selected diffusion-weighted volumes. Note the different colour scales. (*right*) The shell-wise SNR across all acquired *b*-values. The dots represent the estimate of SNR at the subject level, and the box plot represents the median and interquartile range of SNR per given protocol at a group level (*p < 0.05, **p < 0.01).

Figure 3 highlights the improvement in SNR achieved by the use of higher gradient strength, particularly evident at higher *b*-values. The estimated median values and interquartile range (IQR) of SNR demonstrate a notable improvement for each *b*-value at shorter TE. Although our sample size was limited, statistical comparisons between the three protocols revealed significant differences, including for the highest *b*-value (*b* = 3 ms/μm^2^).

### 3.2 Across-protocol image comparison and contrast evaluation

#### 3.2.1 Healthy controls

**Figure 4:**
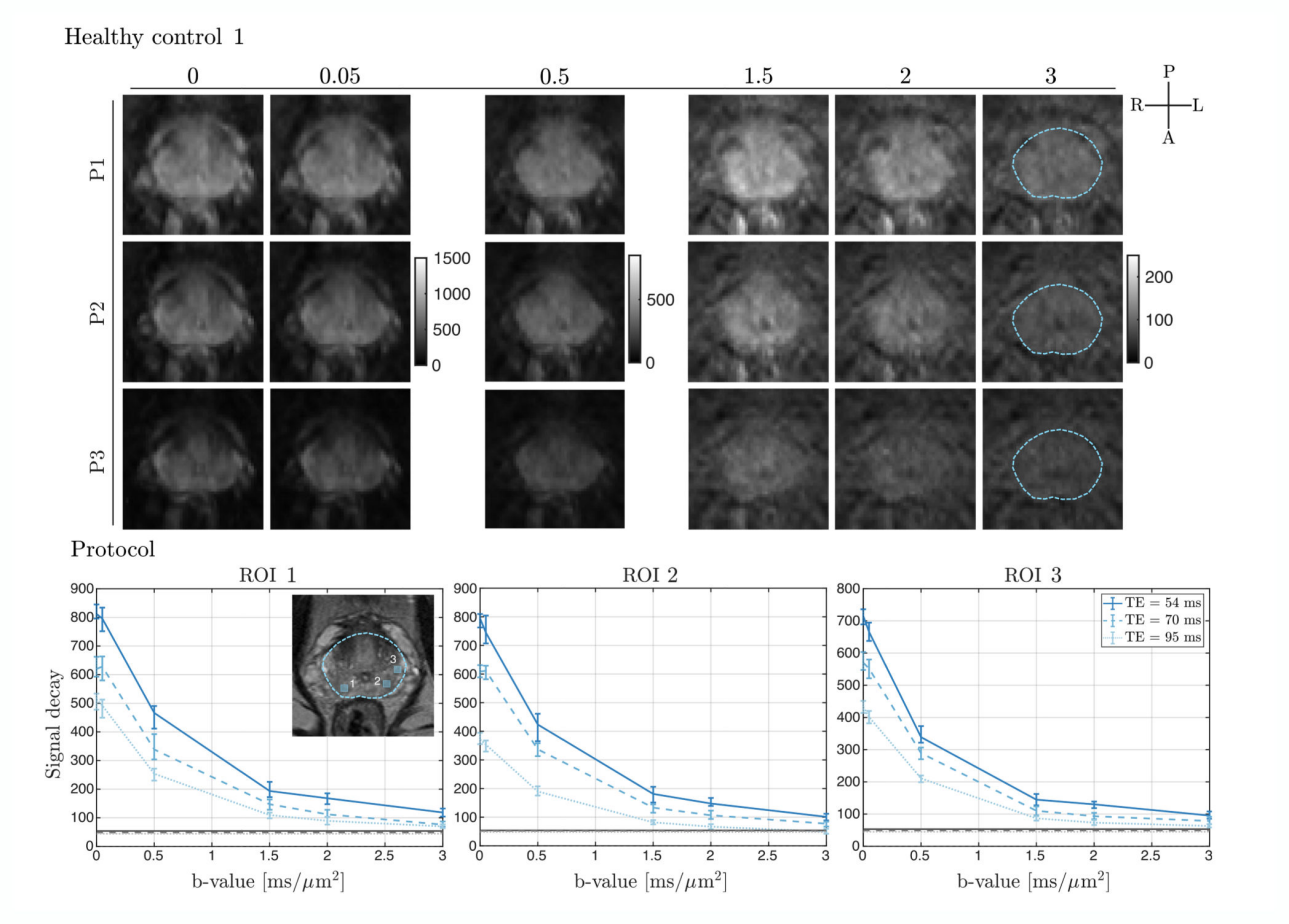
Representative example of a healthy control dataset. The direction-averaged diffusion signals of selected *b*-shell data: 300 mT/m (P1), 80 mT/m (P2) and 40 mT/m (P3) systems (*from top to bottom*) are presented for one healthy control. Based on the *T*_2_-weighted image, a prostate mask was drawn and overlaid on the high *b*-value diffusion dataset. The signal decays (median with interquartile range) of the direction-averaged signals from ROIs (boxes) drawn in the prostate gland are shown. The grey lines represent the mean of the estimated noise floor in each ROI^21^; in most cases, these lines visually overlap.

Figure 4 (*top*) shows the direction-averaged dMRI-images as a function of *b*-value for one healthy control. The signal in prostate decays substantially at *b*-value > 1.5 ms/μm^2^ acquired at the longest TE (P3), whereas for the protocols at shorter TE (specifically *G*_max_ ≈ 300 mT/m, P1), there is residual signal present above the noise floor at *b*-value = 3 ms/μm^2^. This is also evident in the ROI-based signal decay curves (*bottom*), with an estimate of the noise floor. This was true across ROIs [two contralateral regions in the prostate (ROI 1 and ROI 2) and the third area (ROI 3) in the PZ more anteriorly].

#### 3.2.2 Prostate cancer patients

**Figure 5:**
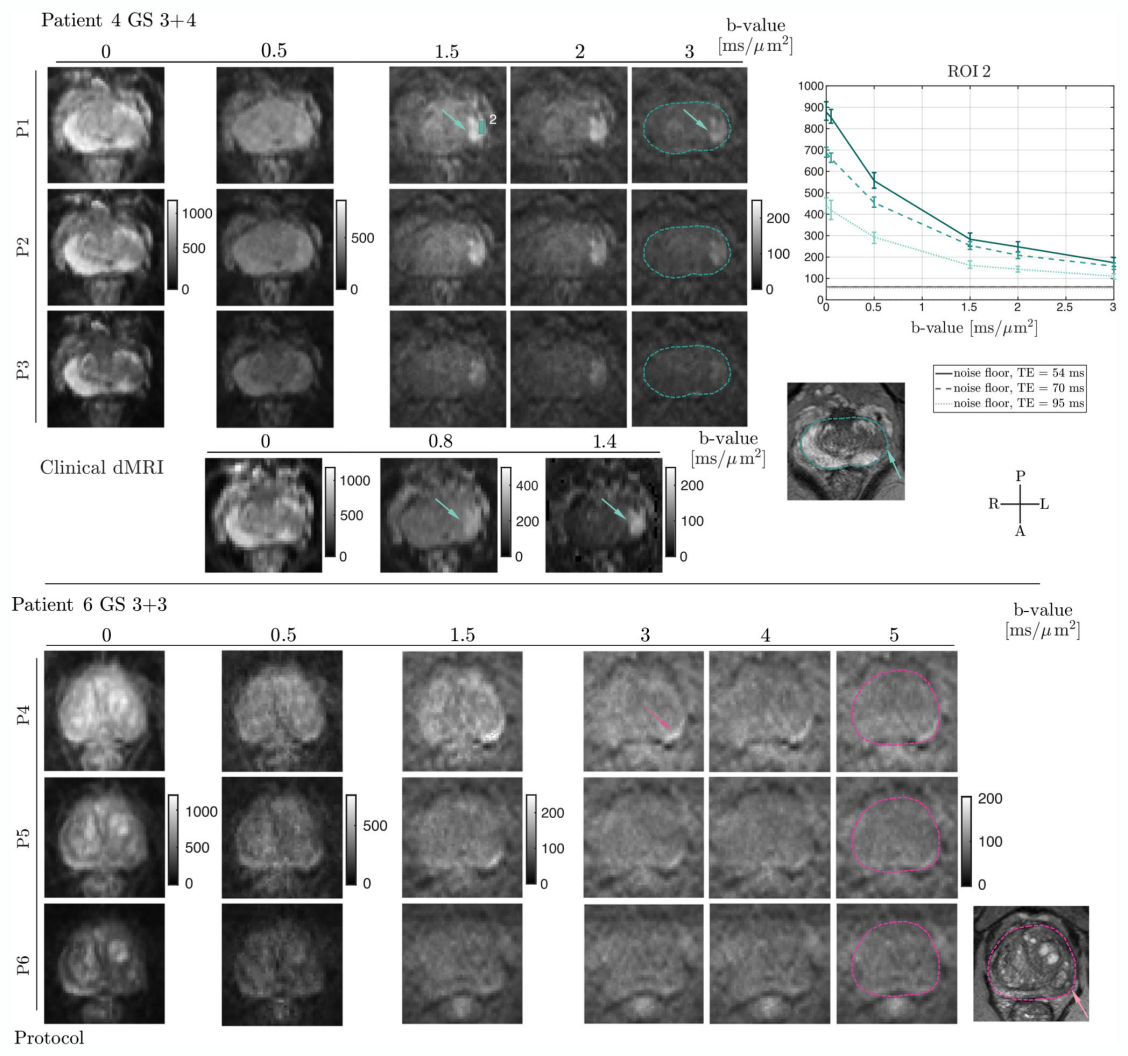
Representative example of patient datasets [P1−P3 protocols (*top*), P4−P6 protocols with ultra-high *b*-value (*bottom*)]. The direction-averaged diffusion signals of the selected *b*-shell data are presented for two patients. As in the case of healthy controls, a prostate mask was drawn on a *T*_2_-weighted image and overlaid on the high *b*-value (3 or 5 ms/μm^2^) diffusion dataset. For Patient 4, the clinical-like dMRI scan and the signal decays (median with interquartile range) of the direction-averaged signals from ROIs (boxes) drawn in the cancerous lesions (depicted by arrows) are shown. The grey lines represent the mean of the estimated noise floor in each ROI^21^; in most cases, these lines visually overlap.

In patients (Figure 5), a visual improvement in contrast between lesions and healthy tissue is observed when using the protocol at the shortest TE (P1). This improvement is corroborated by the quantitative increase in signal intensity in ROIs at *b* = 3 ms/μm^2^ for all investigated GS. Furthermore, Figure 5 (*bottom*) presents the feasibility of increasing the *b*-value up to 5 ms/μm^2^ (protocols P4−P6): Although the signals from the datasets acquired with clinical gradient strength (80 mT/m P5 and 40 mT/m P6) approach the noise floor at 1−3 ms/μm^2^, the dataset acquired with 300 mT/m gradient strength (P4) shows residual signal at the highest *b*-values (4.5−5 ms/μm^2^).

**Figure 6:**
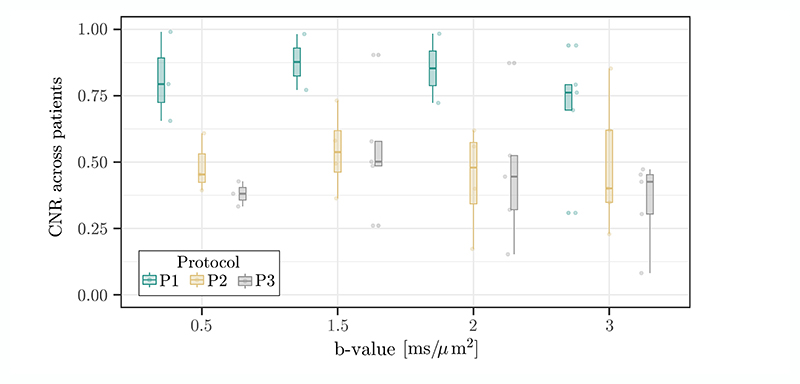
Boxplots of CNR from clinical cohort scanned with P1−P3 protocols for four selected high *b*-values. Dots represent CNR at the subject level, and the boxplot represents the median and interquartile range per given protocol at a group level.

CNR results are shown in Figure 6, highlighting the highest CNR for the strong-gradient P1 protocol across all *b*-values. Within each protocol, the CNR is the highest at *b*-value = 1.5 ms/μm^2^, whereas the biggest difference between protocols is observed at *b*-value = 2 ms/μm^2^ for P1 versus P2 and *b*-value = 0.5 and 2 ms/μm^2^ for P1 versus P3. The linear mixed-effects test confirmed a statistically significant effect of the protocol, including the evaluation of the effect within each of the *b*-values. However, pairwise comparisons did not produce statistically significant results, probably due to the small number of subjects included.

### 3.3 Diagnostic utility of dMRI protocols

All dMRI data were considered of diagnostic quality with the highest average ‘Quality index’ for combinations of data that included *b*-values of 0 and 0.5 ms/μm^2^ (P1: 3.6 ± 0.55, P2: 3.8 ± 0.45, P3: 3.6 ± 0.89). For combinations with higher *b*-values (1.5 and 2 ms/μm^2^), P1 and P2 protocols were scored as equally good or better than P3, without a clear preference for P1 over P2. The image acquired with P1 and *b*-value 0.5 ms/μm^2^ consistently received high scores for diagnostic quality compared with P2 and P3.

In the assessment of the demarcation of ‘zonal anatomy’ combinations with *b*-values of 0 and 0.5 ms/μm^2^ were scored above average in the P1−P3 protocols (3.2 ± 0.45 for all protocols), with below-average scores for the remaining combinations. The evaluated ‘distortions’ were the most prominent in combinations in P3 (3.4 ± 0.89, 2.6 ± 0.55, 1.6 ± 0.55, respectively for 0 and 0.5 ms/μm^2^, 0 and 1.5 ms/μm^2^, 0 and 2 ms/μm^2^). A similar extent of distortions was reported for combinations of data from P1 and P2, with P1 scoring higher for combinations that included higher *b*-values (P1: 2.4 ± 1.14, and 2.2 ± 0.45, vs. P2: 2.2 ± 0.45, and 2 ± 0.71, for 0 and 1.5 ms/μm^2^, 0 and 2 ms/μm^2^, respectively).

In the side-by-side comparison of P1−P3, P1 was generally preferred with two cases where P2 was considered an acceptable alternative. The preference for P1 was reported to be due to its improved diffusion contrast, manifested as a higher intensity of the signal within the prostate gland compared with surrounding tissue at ≥ 0.5 ms/μm^2^ *b*-values.

### 3.4 Quantitative parametric maps from DKI

**Figure 7:**
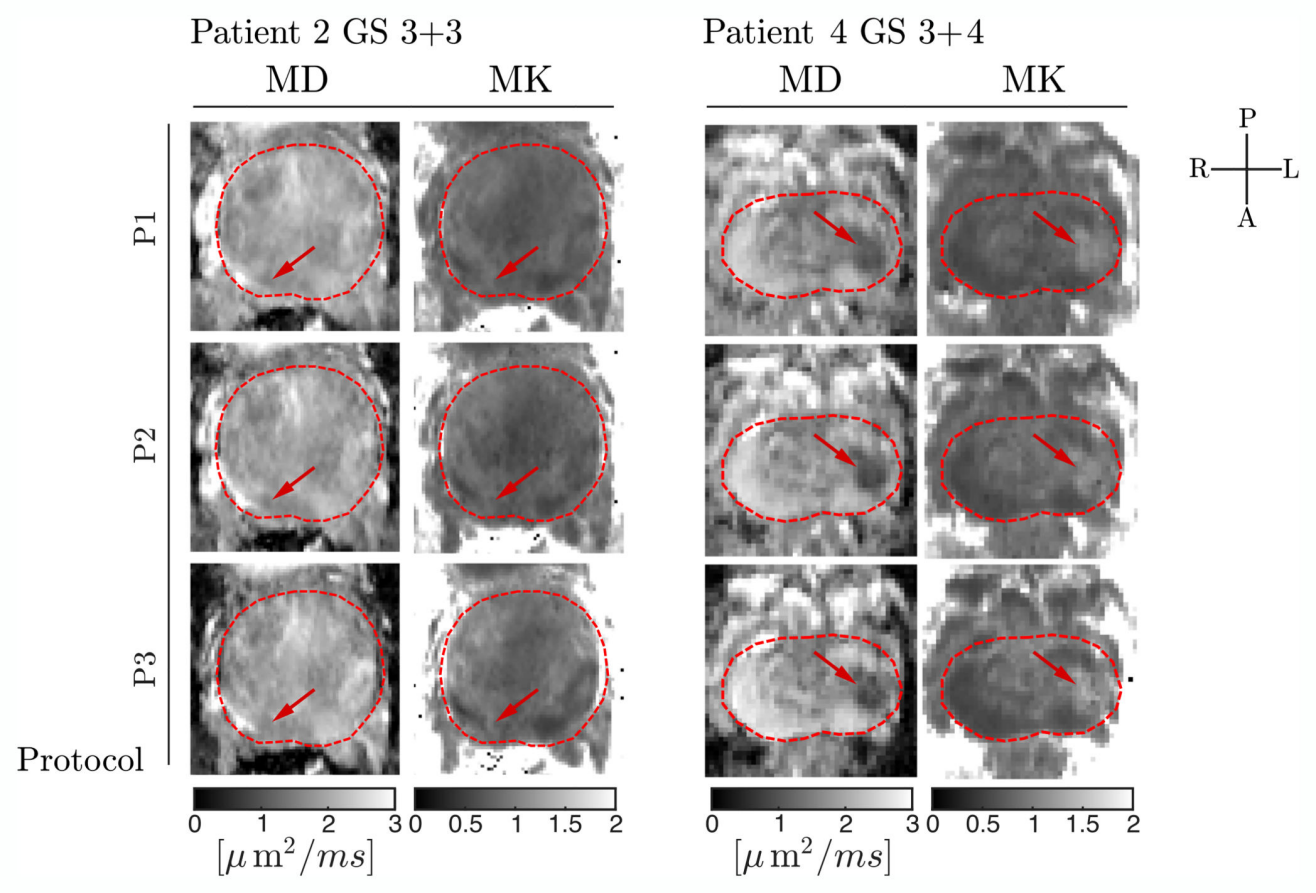
Quantitative parameter maps from the diffusion kurtosis imaging (DKI) fit. Mean diffusivity (MD) and mean kurtosis (MK) maps were estimated from data from two patients (P1−P3 protocols). The cancerous lesions are indicated with red arrows.

The DKI parameter maps (Figure 7) show differences presumably due to different diffusion time and relaxation effects across P1−P3. The median [interquartile range] values across subjects for MD: 1.54 [1.26, 1.64], 1.42 [1.23, 1.61], 1.51 [1.20, 1.73] μm^2^/ms for P1, P2 and P3 protocols, respectively, in cancerous, and 1.94 [1.77, 2.31], 1.92 [1.90, 2.24], 2.09 [2.03, 2.49] μm^2^/ms for P1, P2 and P3 protocols, respectively, in healthy tissue, and MK: 0.99 [0.96, 1.02], 1.02 [0.89, 1.05], 1.04 [0.83, 1.20] for P1, P2 and P3 protocols, respectively, in cancerous, and 0.75 [0.69, 0.77], 0.74 [0.70, 0.82], 0.77 [0.68, 0.80] for P1, P2 and P3 protocols, respectively, in healthy tissue. Pairwise comparisons of the differences between lesions and healthy tissue of MD or MK parameters did not produce statistically significant results. For P1, the maps are more homogeneous in the lesion compared with P2 and P3 and also show the lowest estimates of MD and MK in the prostate. The best contrast between cancerous lesions and healthy tissue in DKI-based maps is observed in P3 (e.g., Patient 4). However, quantitatively, the elevated MD in healthy tissue and increased MK in the cancerous lesions obtained from the data acquired at longer TEs are likely biased due to the lower SNR of the data.

## 4 Discussion

This study shows the feasibility of leveraging the gradient capabilities of a 300 mT/m whole-body system for prostate imaging in healthy subjects and patients diagnosed with PCa without reported adverse effects such as PNS or visual disturbance.^19^ This manuscript has explored the remaining signal at increasingly higher *b*-values, which are commonly off-limits with clinical hardware because of the necessary long TEs, and assessed SNR, CNR and artefacts of measurements in this new regime.

### 4.1 Signal and contrast

The results show that higher *b*-values (≥ 0.5 ms/μm^2^) at shorter TE (and diffusion times) improve the SNR and conspicuity of cancerous lesions in suspected clinically relevant prostate cancer. The radiologist rated images with *b* = 0.5 ms/μm^2^ as having the highest diagnostic quality and utility, which is in apparent contrast with the PI-RADS diagnostic scheme mandating high *b*-values and the argument of using strong gradients. However, the quantitative CNR (Figure 6) is the highest for the strong-gradient protocol (P1) at *b*-value = 1.5 ms/μm^2^, and P1 CNR is notably always higher regardless of the *b*-value compared with any *b*-value acquired with P2 and P3. Visually, image quality is naturally expected to be better at low *b*-values given the improved signal and anatomical detail. In addition, the study cohort has low-grade cancers (3 + 3 and 3 + 4 Gleason score), some of which are subtle compared with background prostate tissue, and higher *b*-values may provide additional advantages in higher-grade cancers.

Conventional prostate imaging uses a maximum *b*-value = 0.8 ms/μm^2^ (following the PI-RADS guidelines) at prolonged TEs. Other researchers used the following combinations of *b-*values and TE for their acquisition: 3 ms/μm^2^ at TE = 90 ms,^30^1.5 ms/μm^2^ at TE = 100 ms,^31^ 1.2 ms/μm^2^ at TE = 148 ms,^32^ 1.5 ms/μm^2^ at TE = 120 ms^7^ and 1 ms/μm^2^ at TE = 74 ms,^33^ among others. In our imaging setup, we acquired data at high *b*-value = 3 ms/μm^2^ at TE = 54 ms. With the availability of new measurement regimes (e.g., the larger range of accessible TE, diffusion-time and *b*-value), the question of which value *b*-value provides the best contrast deserves further investigation as it is dependent on the microstructure and compartmental relaxation (which determines the weighting of compartments at a given TE), among others. Hence, strong gradients provide further opportunities to optimise these parameters to maximise contrast and distinguish low-grade from high-grade lesions.

### 4.2 Artefact corrections

dMRI suffers from artefacts (e.g., signal pile-ups or dropouts and image geometrical distortions) originating from sources such as eddy currents and gradient non-uniformities.^34^ These effects may be amplified with strong gradients. Off-resonance fields caused by magnetic susceptibility differences may vary more in the abdomen compared with, for example, the brain, as different tissue types are contained within the field of view, and interactions between susceptibility effects, eddy currents, gradient non-uniformities and motion are hard to disentangle, which may diminish the expected benefits of strong gradients.

#### 4.2.1 Susceptibility artefacts

No severe susceptibility artefacts due to gas in the rectum were observed in both groups and could be corrected using blip-up/blip-down field map estimation.^23^ However, the correction used only *b* = 0 ms/μm^2^ images, which may impact the accuracy of the estimated deformation field and intensity.^35^ Nevertheless, the corrected images (Figures 4 and 5) showed improved alignment with the *T*_2_-weighted image. Alternatively, other methods can be used to correct for this artefact, either using a separate gradient echo scan^36^ or via custom model-based reconstruction framework^37^ requiring complete dMRI data in both blip-up and blip-down direction. Strong gradients combined with high slew rates have been used to ameliorate susceptibility artefacts,^38^ although PNS prevents to leverage the full whole-body 300-mT/m gradient strength for EPI readout. Here, the readout was kept the same across protocols for comparison, and future studies could leverage the faster EPI readout for further gains.

#### 4.2.2 Eddy current-induced distortions

Qualitative evaluation across subjects revealed a greater, and repeatable, pattern of misalignment between non-diffusion weighted images in P1. This is potentially due to long-time EC, since a shift of the entire image along the PE direction is caused by slice-direction or *B*_0_ EC in EPI-based acquisitions; *B*_0_ EC in the Connectom system have longer decay times (data unpublished) than the reported ∼260 ms.^39^ We hypothesise that dMRI data acquired with P1 are the most affected by EC as the effects of eddy currents scale with the gradient amplitude.^39,40^

A common method to correct EC-induced artefacts in brain dMRI is combined predictive modelling with standard registration techniques.^41^ However, image registration-based methods may not perform satisfactorily in body applications because of lower SNR and less apparent contrast within the organ compared with the brain. Therefore, we adopted a minimal processing approach and plan to further evaluate EC correction in future work. Alternative approaches include EC-compensated diffusion gradients and field monitoring, but their evaluation was beyond the scope of this study.

#### 4.2.3 Gradient non-uniformities

Gradient uniformity is often sacrificed in the design of ultra-strong whole-body or head-only gradient systems.^11,42^ Discrepancies between effective and expected gradients emerge as geometric image deformations, with associated signal intensity deviations, and discrepancies from the intended diffusion encoding.

Although the prostate is a relatively small gland, it is crucial to address the effects of gradient non-uniformities before any further quantitative dMRI analysis as they affect all dMRI images including low *b*-values.^43^ Consequently, spatially varying *b*-matrices, that is, each voxel having a unique set of *b*-values and gradient directions, have to be considered to limit biases in estimates.^25^ The effect of gradient non-uniformities has been mostly investigated in the brain but can also hamper quantitative and cross-centre studies in the prostate (please see Supporting Information SI2).

#### 4.2.4 Motion

Subject motion can lead to signal dropouts and image misalignment. This study did not use hypotonic agents to control peristalsis nor performed breath-hold acquisitions. The PI-RADS v2 guidelines^44^ do not specify patient preparation, and a diet may not be necessary,^45^ but it can be beneficial in reducing intestinal activity and thus distortions near the PZ-rectal wall.

#### 4.2.5 Rician bias

dMRI was reconstructed using an adaptive combine approach,^46^ and therefore, the magnitude data should adhere to a Rician distribution. The noise floor observed at low SNR propagates in all diffusion measures if not corrected for before fitting or not taken into account during model fitting.^28^ Strong gradients enable higher SNR for a given *b*-value and reduce the impact of Rician bias, for example, in our case, as observed in the signal decays (Figures 4 and 5) and in DKI-based maps from acquisition at low gradient amplitude/long TE (Figure 7). Alternatively, postprocessing with the use of complex data^21^ or acquisition of the data with alternative receiver coils (e.g., endo-rectal coils)^47^ could further reduce bias.

### 4.3 Study limitations

This feasibility study had small sample sizes, and groups were not demographically matched. The protocol was designed to obtain the highest *b*value per TE for three different gradient strengths, disregarding diffusion time dependence and transverse relaxation effects. Although this improves the contrast between the lesion and healthy tissue in diffusion-weighted images (Figure 5 and 6), the contrast in DKI maps is not necessarily enhanced (Figure 7), which may be due to differences in TE or diffusion-time across protocols. This causes compartments to be weighted differently if they exhibit distinct *T*_2_ and time dependence.

The utility of novel scans was assessed by one radiologist, and a more extensive radiological evaluation is planned in future work. In addition, the study cohort has low-grade cancers, and thus, we could foresee less use of higher *b*-values. Nevertheless, the image quality is satisfactory, and we envision that these images at higher *b*-values would show more gain in higher cancer grades because of positive correlation with increasing restrictions.^48^

This work was exploratory, and future work will aim to establish clinically feasible minimal protocols for the next-generation MRI scanners.

### 4.4 Outlook

With improved gradient hardware becoming more readily available also commercially, this study highlights the potential of powerful gradients in prostate cancer diagnosis and treatment planning. A recent study recognised that changes in the epithelium, stroma and lumen signal fractions estimated from MRI data correlate more strongly with Gleason grade than any cellularity metrics from histological data (e.g., nuclear count).^48^ The observation that individual dMRI images contain useful contrast suggests that they offer new possibilities as a part of multi-*b*-value protocols for estimating quantitative parameters of tissue composition, which have been shown to be uniquely able to differentiate clinical significant from insignificant cancers even at clinical gradient strengths.^9^ Leveraging strong gradients in prostate imaging will allow better estimates of the parameters in biophysical models (e.g., VERDICT^8^) and more advanced phenomenological signal representations more indirectly related to microstructural features. In addition to facilitating higher *b*-values, strong gradients allow for the variation of TE and diffusion time over a much larger range for a given maximum *b*-value than clinical gradients, which enables the additional estimation of compartmental relaxations and time dependencies,^31,49,50^ among others, and thus provides potential novel quantitative biomarkers.

## 5 Conclusions

This work addresses the main technical impediment to clinical dMRI in the prostate: poor image contrast and low SNR at high *b*-values. Strong gradients compared with the more widely available clinical low-amplitude gradients outweigh their inherent drawbacks as a significant gain in CNR in early prostate cancer is obtained. Further development of more advanced microstructural acquisition sequences and analysis methods dedicated to the unique data provided by strong gradients should lead to a powerful non-invasive diagnostic tool that will improve the specificity and/or sensitivity of MRI for the detection and microstructural characterisation of prostate cancer.

## Supplementary Material

Supporting information

## Figures and Tables

**Table 1:** Clinical cohort data overview.

ID	Age	Years on AS	PSA (ng/mL)	PIRADS	Tumour location	Tumour size	Gleason score
PT 1	74	3	5.2	4 + 3	Right posterolateral PZ at the apex, left anterior PZ at the apex	4 mm, 3 mm	3 + 3
PT 2	68	3	6	3	Right posteriolateral PZ at the base	7 mm	3 + 3
PT 3	72	2	12.8	5 + 3	Left posterior and lateral PZ, right posteriolateral PZ	20 mm, 9 mm	3 + 4
PT 4	67	5	4.7	4	Left PZ at the base and midgland	13 mm	3 + 4
PT 5	68	4	4.3	3	Left lateral PZ, right lateral PZ at midgland level	4 mm, 5 mm	3 + 4
PT 6	72	3	6	No record	Left PZ at the base and midgland	No record	3 + 3

*Note*: The “Years on AS” is defined based on the year of the earliest mp-MRI scan and biopsy. The clinical information (apart from age) is dated based on the latest clinical records as of the end-date of this study. Abbreviations: AS, active surveillance; PSA, prostate-specific antigen; PT, patient.

## Data Availability

Due to ethical concerns, supporting data cannot be made openly available. Data can be provided upon request and signature of data sharing agreement. Please contact cubric@cardiff.ac.uk in the first instance.
