# Supplementary material for: Diffusion MRI in prostate cancer with ultra-strong whole-body gradients
Source: NMR Biomed. Author manuscript; Available in PMC 2025 Mar 13. (PMC7617477; doi:10.1002/nbm.5229)
Supplement: Supporting information [file EMS203647-supplement-Supporting_information.zip › nbm5229-sup-0001-nmrinbiomed_prostate_300mtm_dmri_molendowska_main_manuscript_si_r1_revised.docx]

Supporting Information

SI1: Prostate Tissue Simulations

The benefits of ultra-strong gradients in the context of imaging different microstructural compartments of prostate tissue are shown in Figure 1C. Signals were simulated for benign and cancerous tissue based on a three pool model^8^ composed of the stroma, epithelium and lumen with different transverse relaxation, *T*_2_, and diffusion, *D*, properties. For simulations, we used SNR = 25 and SNR = 30 for healthy and cancerous tissue, respectively. The SNR was calculated at *b* = 0 ms/µm^2^ at TE = 0 ms as SNR*_b_*_=0, TE_$\neq$_0_ *=* SNR *_b_*_=0, TE=0_ exp(-TE/T_2_) using the SNR estimates from in vivo data and average *T*_2_ values of prostate tissue^51^. The simulations were performed according to the values reported in Zhang et al.^8^ with diffusivities scaled by a factor of 2 to reflect tissue in vivo. Ex vivo estimates of the *T*_2_ values were used. The expected volume fractions of three microenvironments present in the prostate as given in the diagrams *T*_2_ *−D*. Fifteen Rician noise realisations per *b*-value were performed to reflect the number of different gradient directions in our protocols. To allow direct comparisons with in vivo data, we multiplied the simulated normalised signals by the estimated signal *S*_0_ at *b* = 0 ms/µm^2^ and TE = 0 ms using experimental *S*_0_ values at *b* = 0 ms/µm^2^ measured at different TEs (’benign case’ - healthy control 1, ’cancerous case’ - patient 4).

Reducing TE at a given *b*-value significantly increases the signal contribution from the supportive stromal compartment (short *T*_2_-pool of approximately 30 ms with intermediate diffusivity of 1-2 ms/µm^2^) as well as the epithelium. For changes associated with cancerous tissue in which diffusion becomes more restricted, higher *b*-values contain significant information above the noise floor. The simulations resemble the signal decays obtained from in vivo data (see the Results sections and Figures 4 - 5 of the main manuscript).

SI2: Quality Assessment of dMRI Data

dMRI data was collected with 4 consecutive *b* = 0 ms/µm^2^ images acquired at the beginning of the protocol and with additional *b* = 0 ms/µm^2^ interleaved every 6^th^ diffusion direction. The purpose of such an acquisition sampling scheme was to facilitate the evaluation of image shifts or distortions.

During visual data evaluation, the images were not altered (shifted with respect to the 1^st^ shot) in the first few *b* = 0 ms/µm^2^ shots, whereas those were present in volumes acquired after encoding with high diffusion weighting, i.e., high gradient strength. These effects were consistent in 9 subject datasets scanned with P1-P3 protocols. We expect that the distortions that are observed are a result of short- and perhaps long-term EC.

Please refer to Video S1 for visualisation of the effects of the eddy currents in the imaging protocols.

For participants positioned at isocentre, the estimated effective *b*-values inside the prostate deviated from the imposed values by maximally 5% and typically in the range of 0-3%. A deviation in the positioning of +/- 80-100 mm along the *Z*-axis can cause on average 10% and up to 25% deviation in *b*-value (Figure S1).

Video S1. EC effects in *b* = 0 ms/µm^2^ and *b* = 0.5 ms/µm^2^ images in the P1-P3 protocols used in this study. Please note that in the P1 protocol, the shifts or distortions of the images are more prominent than in the acquisitions using lower gradient strengths, such as P2 and P3. These shot-to-shot image misalignment is arising from interaction of long-term or slice-direction EC (*b* = 0 ms/µm^2^) or short-term EC (*b* = 0.5 ms/µm^2^) and, if present, motion.

Figure S1. Distribution plots of the deviations of *b*-values caused by gradient non-uniformities for all *b*-values in data from four selected subjects (Patient 1 - positioned in the isocentre, Patient 2 - positioned off-centre by approximately 8 cm, Healthy control 1 - positioned off-centre by approximately -10 cm, Healthy control 2 - positioned at isocentre).

**REFERENCES**

8. Zhang, Z.; Wu, H. H.; Priester, A.; Magyar, C.; Afshari Mirak, S.; Shakeri, S.; Mohammadian Bajgiran, A.; Hosseiny, M.; Azadikhah, A.; Sung, K., et al. Prostate microstructure in prostate cancer using 3-T MRI with diffusion-relaxation correlation spectrum imaging: Validation with whole-mount digital histopathology. *Radiology* 2020, *296*, 348–355.

51. Lee, C. H. Quantitative T2-mapping using MRI for detection of prostate malignancy: A systematic review of the literature. *Acta Radiologica* 2019, 60, 1181–1189.
