# Supplementary figures and images for: Diffusion MRI in prostate cancer with ultra-strong whole-body gradients
Source: NMR Biomed. Author manuscript; Available in PMC 2025 Mar 13. (PMC7617477; doi:10.1002/nbm.5229)

# Effects of gradient non-uniformities on b-values

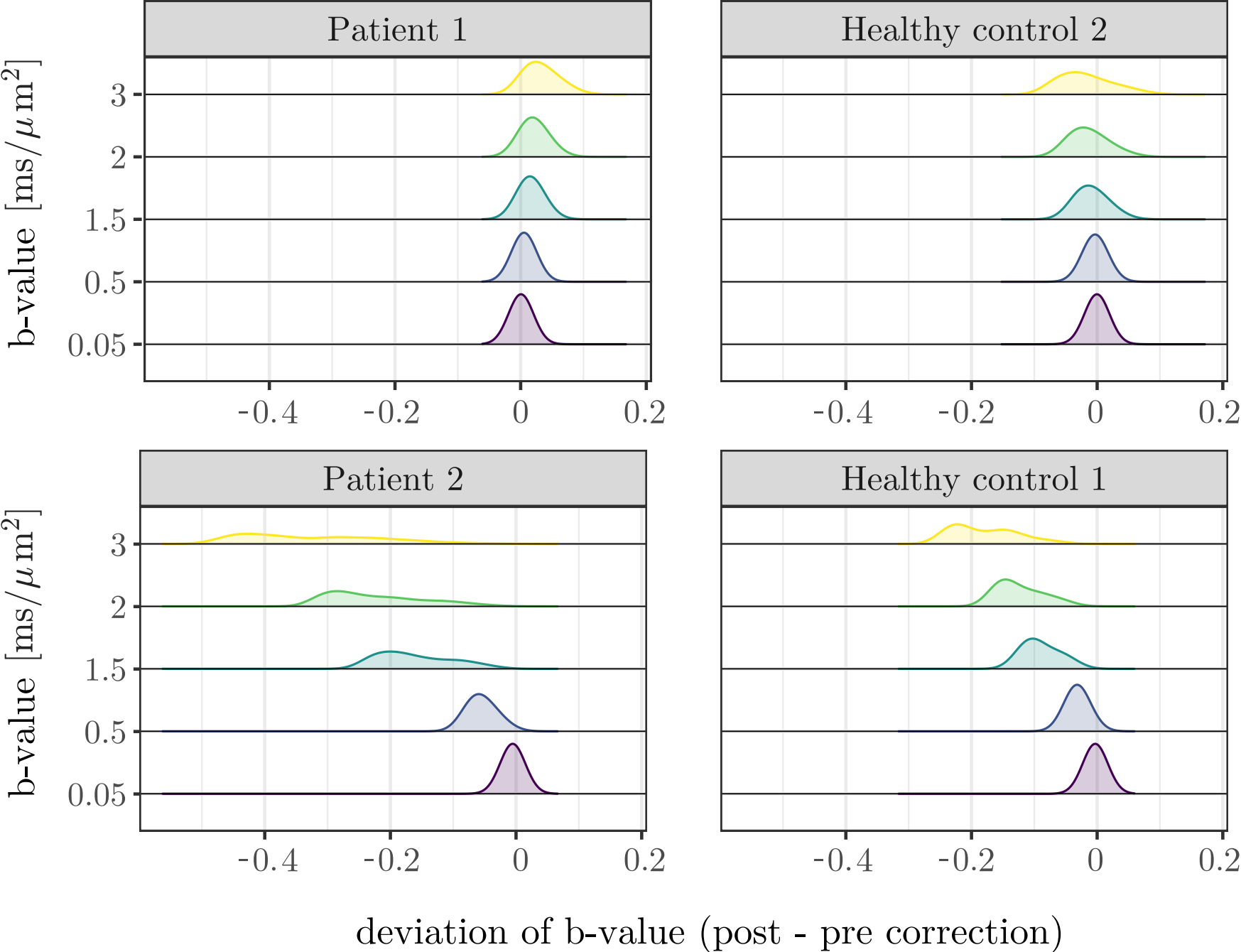

Supplement: Supporting information [file EMS203647-supplement-Supporting_information.zip › nbm5229-sup-0002-6_supp_figure_1_gnl_correction_si_r1.pdf]
